# Calcium silicate-based cements cause environmental stiffness and show diverse potential to induce osteogenesis in human osteoblastic cells

**DOI:** 10.1038/s41598-021-96353-0

**Published:** 2021-08-18

**Authors:** Marcos Coelho Santiago, Ana Lívia Gomes-Cornélio, Laudimar Alves de Oliveira, Mario Tanomaru-Filho, Loise Pedrosa Salles

**Affiliations:** 1 Post-Graduation Program in Dentistry, Faculty of Health Sciences, University of Brasilia (UnB), Campus Universitário Darcy Ribeiro, Asa Norte, Brasília, DF 70910-900 Brazil; 2 Department of Dentistry, Hospital das Forças Armadas (HFA), Cruzeiro Novo, Brasília, DF 70658-900 Brazil; 3 Department of Dentistry, Faculty of Health Sciences, University of Brasilia (UnB), Campus Universitário Darcy Ribeiro, Asa Norte, Brasília, DF 70910- 900 Brazil; 4 Department of Restorative Dentistry, Araraquara Dental School, Universidade Estadual Paulista Júlio de Mesquita Filho, Campus de Araraquara, Araraquara, SP 14801-903 Brazil; 5 Department of Dentistry, Faculty of Health Sciences, University of Brasília (UnB), Campus Universitário Darcy Ribeiro, Asa Norte, Brasília, DF 70910-900 Brazil

**Keywords:** Dentistry, Endodontics, Root canal treatment, Biomaterials - cells

## Abstract

Calcium silicate-based cements differ markedly in their radiopacifiers and the presence of calcium sulfate, aluminates, carbonates and other components that can affect their biological properties. This study aimed to compare the biological properties of six calcium silicate cements in human osteoblastic cell culture (Saos-2 cells): Bio-C Repair (Bio-C), PBS HP (PBS-HP), Biodentine (Biodentine), MTA Repair HP (MTA-HP), NeoMTA Plus (NeoMTA-P), and ProRoot MTA (ProRoot). After exposure to these materials, the cells were analyzed by MTT, wound healing, cell migration, and alkaline phosphatase activity (ALP) assays, real-time PCR (qPCR) analysis of the osteogenesis markers (osteocalcin or bone gamma-carboxyglutamate protein, *BGLAP*; alkaline phosphatase, *ALPL*; bone sialoprotein or secreted phosphoprotein 1, *BNSP*), and alizarin red staining (ARS). Curiously, the migration rates were low 24–48 h after exposure to the materials, despite the cells showing ideal rates of viability. The advanced and intermediate cell differentiation markers *BGLAP* and *BNSP* were overexpressed in the Bio-C, MTA-HP, and ProRoot groups. Only the Biodentine group showed *ALPL* overexpression, a marker of initial differentiation. However, the enzymatic activity was high in all groups except Biodentine. The mineralization area was significantly large in the NeoMTA-P, ProRoot, PBS-HP, MTA-HP, and Bio-C groups. The results showed that cellular environmental stiffness, which impairs cell mobility and diverse patterns of osteogenesis marker expression, is a consequence of cement exposure. Environmental stiffness indicates chemical and physical stimuli in the microenvironment; for instance, the release of cement compounds contributes to calcium phosphate matrix formation with diverse stiffnesses, which could be essential or detrimental for the migration and differentiation of osteoblastic cells. Cells exposed to Bio-C, PBS-HP, ProRoot, NeoMTA-P, and MTA-HP seemed to enter the advanced or intermediate differentiation phases early, which is indicative of the diverse potential of cements to induce osteogenesis. Cements that quickly stimulate osteoblast differentiation may be ideal for reparative and regenerative purposes since they promptly lead to dentin or bone deposition.

## Introduction

Repairing root perforations, root resorptions, and open apexes represents a challenge for endodontists, mainly because these conditions demand the formation of mineralized tissue^1^. This challenge has led to the evolution of calcium silicate-based cements with increased biocompatibility and bioactivity^1^. In this context, mineral trioxide aggregate (MTA) represents a milestone in the use of calcium silicate cements for reparative endodontic treatment^1^.

MTA can be considered the precursor of new calcium silicate-based cements^2^. The first MTA formulation was composed of calcium silicates, bismuth oxide, calcium carbonate, calcium sulfate, and calcium aluminate^1^. MTA (Angelus) replaced radiopacified bismuth oxide (Bi_2_O_3_) with calcium tungstate (CaWO_4_)^2^. The results presented in clinical trials led to MTA becoming the material of choice for retrograde fillings and the treatment of root perforations^3^. However, MTA still presents disadvantages, such as handling difficulties, dental structure discoloration, a fast initial setting time (approximately 30 min), and a long final setting time (up to 10 h), which has prompted the development of new calcium silicate cements^4,5^. These new compositions aim to increase the biocompatibility as well as the flow and release of calcium ions^1,5^. In particular, the release of Ca^2+^ enhances the formation of calcium hydroxide through the chemical reactions of tricalcium and dicalcium silicates during material setting^4^.

Biodentine was one of the first cements indicated as a bioactive substitute for MTA during reparative and regenerative therapy^6^. In general, the new calcium silicate-based cement components are calcium oxides, calcium carbonates, calcium hydroxide, or calcium phosphates, which are added to calcium silicates and radiopacifying agents^7,8^. Biodentine, for example, is composed of tricalcium and dicalcium silicate, with zirconium oxide as the radiopacifying agent^6^. Recently, Rathinam et al.^9^ evaluated the viability and matrix mineralization potential of human dental pulp stem cells (hDPSCs) exposed to Biodentine and ProRoot MTA. This study demonstrated that calcium silicate-based cements alter extracellular and intracellular Ca^2+^ dynamics and trigger hDPC differential gene expression, differentiation, and mineralization potential. ProRoot MTA exposure caused downregulation of bone morphogenetic 2 (*BMP2*) gene expression in hDPSCs and, in contrast, Biodentine stimulated its overexpression^9^. It was also found that the release of Ca^2+^ from Biodentine was higher than that from ProRoot MTA^9^. The specific calcium dynamics of these biomaterials determines their differentiation and mineralization outcomes. Increases in Ca^2+^ dynamics enhances mineralization. Interestingly, the authors confirmed that the frequency, duration, and amplitude of Ca^2+^ transients are essential for increasing the efficiency and specificity of gene expression in dental pulp cells^9^. Another study revealed that a water-based tricalcium silicate cement had greater Ca^2+^ ion release, which is associated with a strong antimicrobial effect but not cytotoxicity in mouse 3T3 cells (a model to study the calcium-mediated actin reset due to physiological changes) or human dental pulp cells^10^. In contrast, studies have indicated cytotoxicity for Biodentine, mainly before it is completely set (fresh leachates) and also according to the concentration^10,11^. Another limitation of Biodentine is the need for specific preparation equipment^11–13^. A ready-to-use calcium silicate-based cement, Bio-C Repair, recently became available for reparative or regenerative endodontic treatments.

The manufacturer claims that the material presents the biological properties of MTA-based cements with easier handling. The composition of Bio-C Repair cement evolved from its precursor MTA Repair HP. Both of these cements are calcium silicate-based; however, MTA Repair HP has calcium tungstate as the radiopacifying agent^14^, while in Bio-C Repair, the radiopacifier is substituted by zirconium oxide. A study evaluating the physical–chemical properties of MTA HP Repair with ProRoot MTA and NeoMTA Plus showed promising results with respect to the setting time, hydration, and Ca^2+^ ion release^14^. Interestingly, NeoMTA Plus is another silicate-based cement, in which its main difference is the use of tantalum oxide (Ta_2_O_5_) as the radiopacifying agent. From 2010 to 2012, there was great interest in new MTA formulations, especially changing the radiopacifying agent to something other than bismuth oxide. The possible interference of these composition changes on the biological properties of the cement must be investigated^14^. PBS HP, composed of dicalcium silicate, mineral oxides, and pozzolan, is a recently proposed cement. PBS HP showed promising results as a graft material in dental implants by maintaining the socket architecture^15^. Histological images showed newly formed fibroblasts, osteoid cells, mineralized bone tissue, and connective tissue encasing PBS HP. Additional information on the properties of PBS HP is still very scarce. The manufacturers indicate the use of Bio C Repair and PBS HP for reparative and regenerative treatments. However, the scientific literature lacks comparative studies evaluating the biological properties of Bio-C Repair and PBS HP with other calcium silicate-based materials. In addition, the literature shows that dental pulp and bone cells respond in diverse ways to different calcium silicate-based cements.

Therefore, this study aimed to evaluate the biocompatibility and osteogenesis-inducing potential of Bio-C Repair (Bio-C), PBS HP (PBS-HP), Biodentine (Biodentine), MTA Repair HP (MTA-HP), NeoMTA Plus (NeoMTA-P) and ProRoot MTA (ProRoot) in cultures of human osteoblastic cells (Saos-2). Saos-2 cells are considered to be a suitable model to study osteoblast differentiation^11,16,17^. Our general null hypothesis was that there was no difference between the cell responses after exposure to these different materials.

## Results

### Cell viability

**Figure 1:**
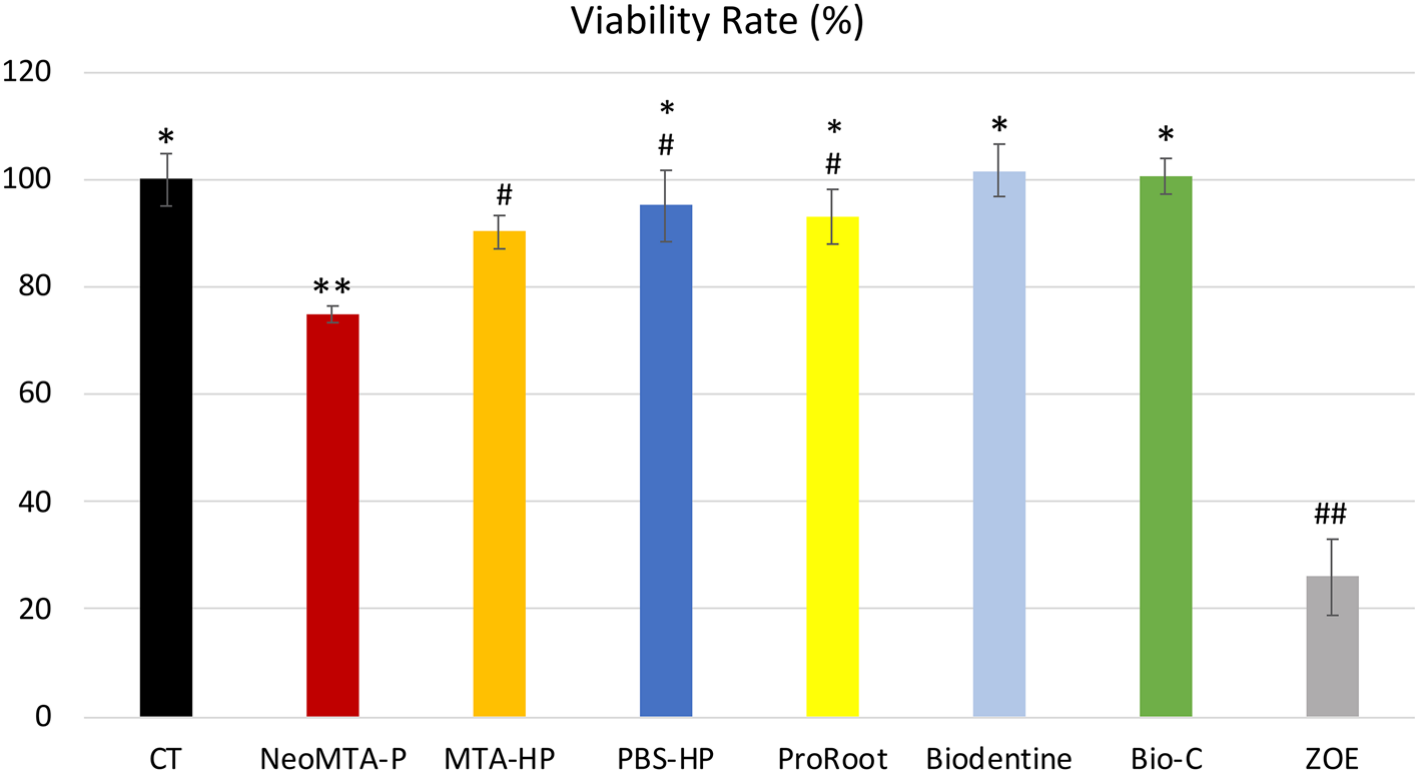
Cell viability rates. CT, positive control group (unexposed cells). NeoMTA-P, MTA-HP, PBS-HP, ProRoot, Biodentine, Bio-C, and ZOE (negative control) were the groups of cells exposed to the respective types of cement. The viability rates of the cells exposed to PBS-HP, ProRoot, Biodentine, and Bio-C were similar to those of the control group. However, NeoMTA-P and MTA-HP exposure led to a significantly lower viability rate than CT. Data are presented as mean ± SD; one-way ANOVA, followed by multiple comparisons post-hoc test with Bonferroni’s test. Symbols on the chart indicate homogeneous groups by Bonferroni’s posttest. *p < 0.005, all groups versus CT; **p < 0.001, comparisons among cement groups; ^#^p < 0.005, comparisons among cement groups; ^##^p < 0.001, ZOE versus all groups.

After 24 h, Saos-2 cells exposed to Bio-C, PBS-HP, ProRoot, and Biodentine showed viability rates statistically similar to that of the control group (CT; 100.65%, 95.3%, 98.5%, 101.78%, and 100.23%, respectively). The cells exposed to NeoMTA-P (75.10%) and MTA-HP (90.36%) showed significantly lower viability rates than CT (Fig. 1). All groups had higher viability rates than ZOE (25.97%).

### Cell migration

**Figure 2:**
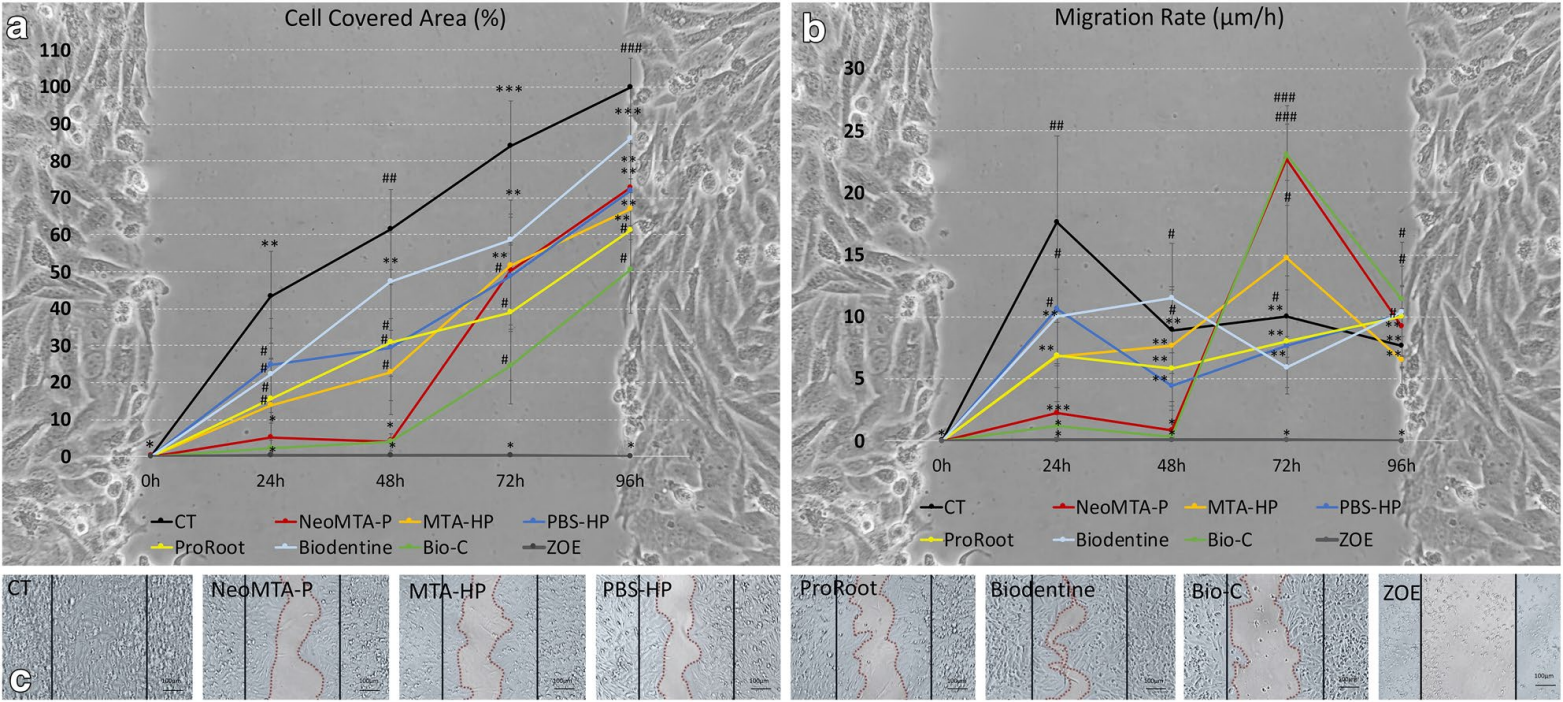
(**a**–**c**) Wound area covered by cells, cell migration rates, and representative images of the cells after 96 h of exposure. The surface areas of the wound covered by the osteoblastic cells exposed to the reparative cements were significantly smaller than the control group of unexposed cells (CT) at all times evaluated (**a**). Impairment of cell migration could be observed at 24 h in the calcium silicate-based cement groups, suggestive of environmental stiffness (**b**). Osteoblastic cells exposed to NeoMTA-P and Bio-C for 24 and 48 h showed a significantly lower migration rate than the other groups, especially when compared to CT. From 48, 72, to 96 h, the migration rates of the osteoblastic cells exposed to MTA-HP, PBS-HP, ProRoot, and Biodentine were similar to that of CT. The cells exposed to NeoMTA-P and Bio-C showed a significant increase in migration rates only after 72 h of exposure (**b**). At 96 h, the unexposed cells (CT) showed complete closure of the wound area. However, the cells exposed to NeoMTA-Plus, MTA-HP, PBS-HP, ProRoot, Biodentine, and Bio-C did not migrate enough to close the wound gap (**c**). Interestingly, the cell morphologies were typical without evident signs of cytotoxicity except for the comparator group (ZOE), which showed dead cells and a completely open area (**c**). Micrographs at ×20, 100 µm bars (**c**). The backgrounds in (**a**,**b**) are representative images of the wound healing at the initial time (T0). Data are presented as mean ± SD of area covered by cells (**a**) and migration rates (**b**) at each exposure time; two-way ANOVA followed by multiple comparisons post-hoc test with Bonferroni’s posttest. Symbols on the charts indicate homogeneous groups by Bonferroni’s posttest. (**a**) *p < 0.001, all groups within the different times of exposure versus CT (0 h); ^#^p < 0.02, cement groups versus each other within the different times of exposure; **p < 0.05, all groups within the different times of exposure versus CT (24 h); ^##^p < 0.03, all groups within the different times of exposure versus CT (48 h); ***p < 0.02, all groups within the different times of exposure versus CT (72 h); and ^###^p < 0.005, all groups within the different times of exposure versus CT (96 h). (**b**) *p < 0.001, all groups within the different times of exposure versus CT (0 h); ^#^p < 0.05, all groups versus each other within the different times of exposure; **p < 0.03, all groups versus each other within the different times of exposure; ^##^p < 0.02, all groups within the different times of exposure versus CT (24 h); ***p < 0.02, NeoMTA-P (24 h) versus all groups within the different times of exposure; and ^###^p < 0.001, NeoMTA-P and Bio-C (72 h) versus all groups within the different times of exposure.

Saos-2 cells exposed to Bio-C and NeoMTA-P showed similar percentages of wound areas, with open spaces at 0, 24, and 48 h (Fig. 2a). The migration rates (Fig. 2b) of the cells exposed to Bio-C and NeoMTA-P were low until 48 h (< 3 µm/h) and only increased after 72 h (> 20 µm/h). The PBS-HP and Biodentine groups showed higher migration rates than the other groups at 24 h but a significantly smaller migration rate than the control group (CT). The migration rates of Bio-C, MTA-HP, and NeoMTA-P exceeded that of the CT only at 72 h. The PBS-HP, Biodentine, and ProRoot groups (> 10 µm/h) exceeded the CT migration rate only at 96 h. The association between the migration rate and exposure time was significant according to the two-way ANOVA (p < 0.05), which indicates that the relationship between the type of cement and the migration rate was dependent on the exposure time (Fig. 2b). From the microscopy observations (Fig. 2c), the control group (CT) was the only group with complete closure of the wound area at 96 h. Interestingly, the cells adjacent to the wound in the calcium silicate cement groups were overconfluent with typical morphologies.

### Gene expression

**Figure 3:**
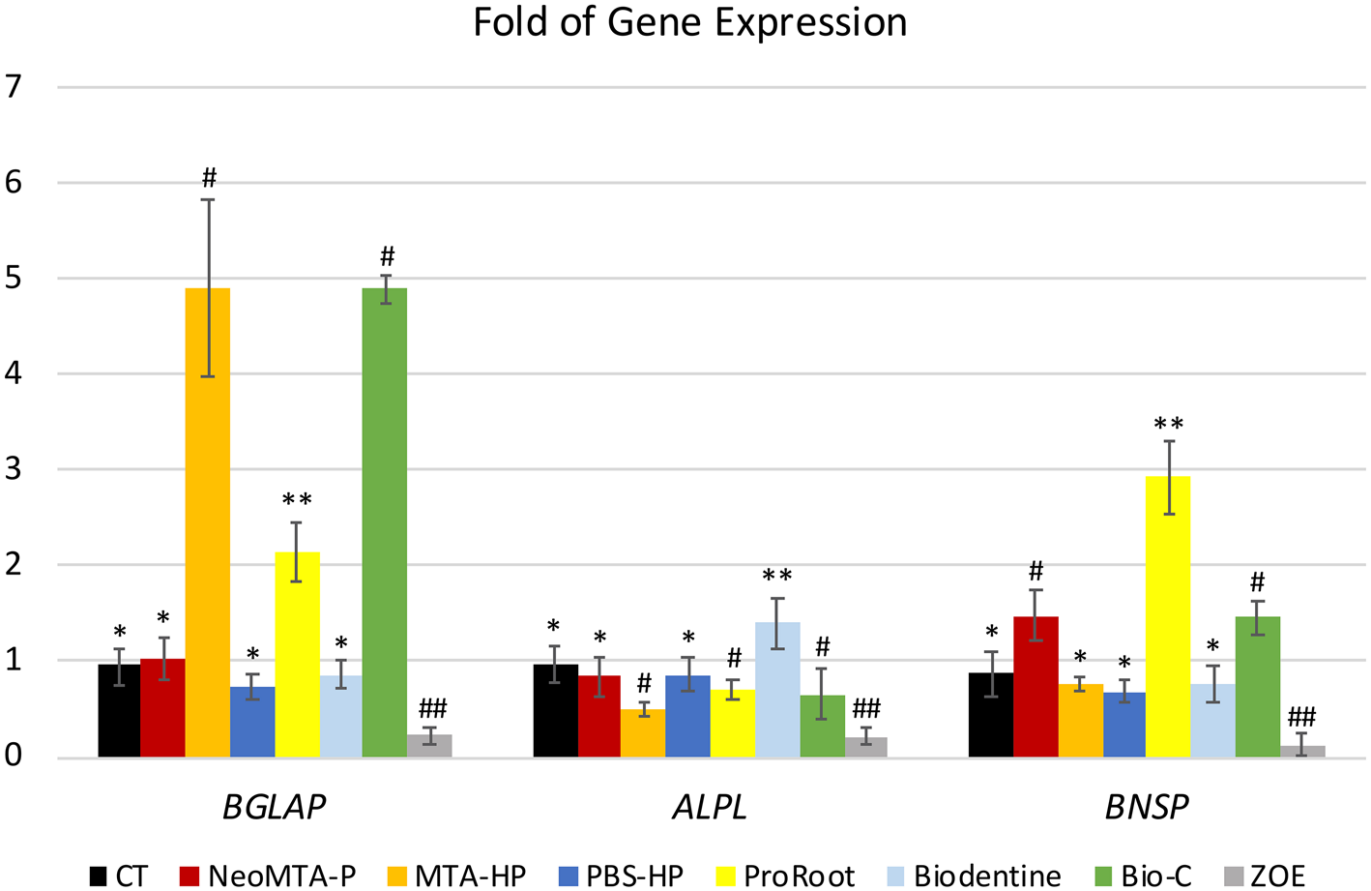
Fold change in expression of the genes *BGLAP*, *ALPL*, and *BSP*. The fold change in the expression of osteocalcin (*BGLAP*) was significantly high in the ProRoot, Bio-C, and MTA-HP groups after 96 h of cell exposure. The bone sialoprotein (*BNSP*) gene was significantly overexpressed in the Bio-C, NeoMTA-P, and ProRoot groups. Only the Biodentine group showed a significantly high expression of alkaline phosphatase (*ALPL*). All genes were inhibited in the ZOE group. Data are presented as mean ± SD; one-way ANOVA, followed by multiple comparisons post-hoc test with Bonferroni’s test. Symbols on the chart indicate homogeneous groups by Bonferroni’s posttest. *p < 0.005, all groups versus CT; **p < 0.001, comparisons among cement groups; ^#^p < 0.002, comparisons among cement groups; ^##^p < 0.003, ZOE versus all groups.

After 96 h of exposure, the fold change in the expression of osteocalcin (*BGLAP*) was significantly high in the ProRoot (2.15 ± 0.30; mean and stdv), Bio-C (4.88 ± 1.60), and MTA-HP (4.89 ± 0.92) groups. The bone sialoprotein (*BNSP*) gene was significantly overexpressed in the Bio-C (1.45 ± 0.32), NeoMTA-P (1.48 ± 0.27), and ProRoot groups (2.92 ± 0.32) compared to the control group. Only the Biodentine group showed higher expression of the alkaline phosphatase gene (*ALPL*) than the CT group. The PBS-HP, Bio-C, NeoMTA-P, and ProRoot groups showed statistically similar inhibition of *ALPL* expression. All of these genes were inhibited in cells exposed to the negative control ZOE (Fig. 3).

### Alkaline phosphatase activity

**Figure 4:**
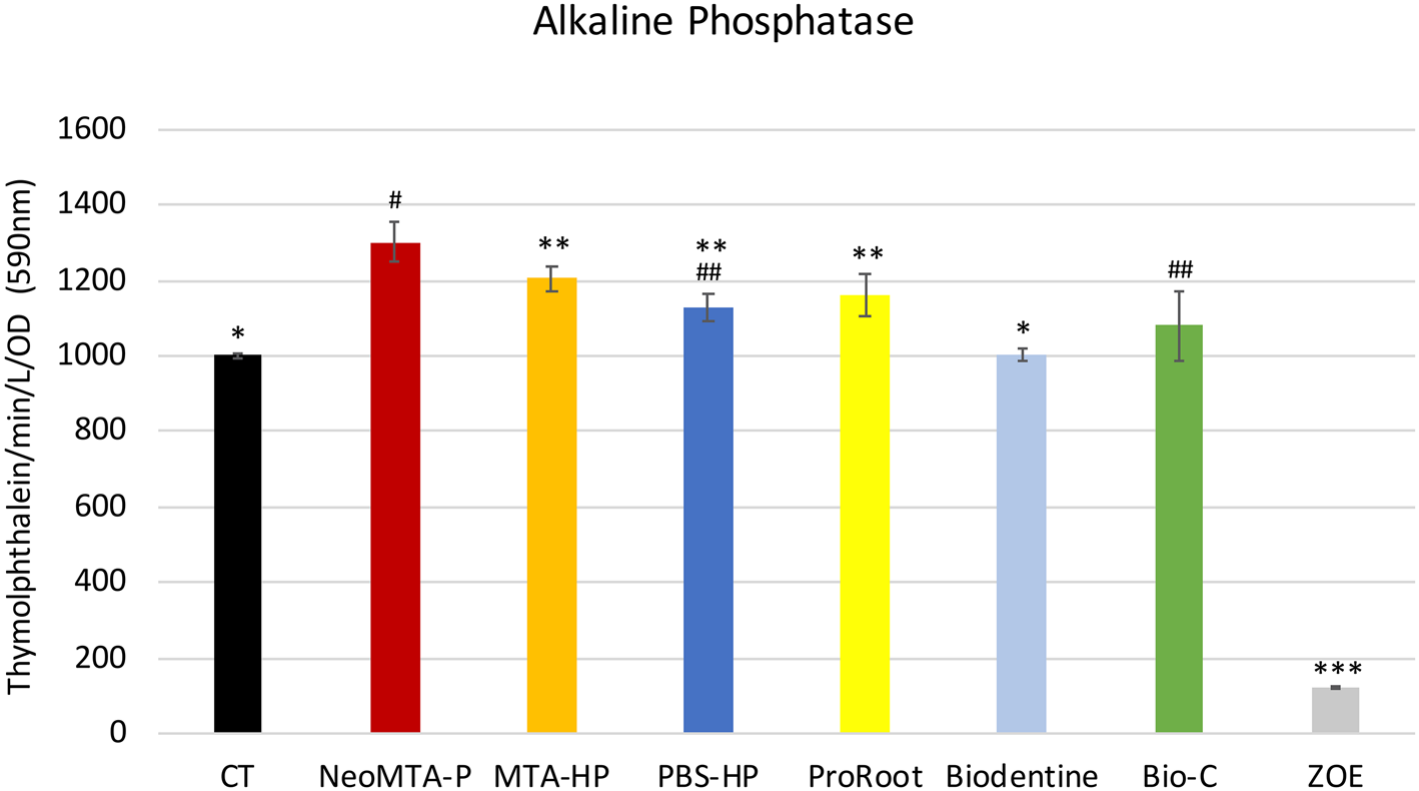
Alkaline phosphatase activity (ALP). The osteoblastic cells showed significantly high ALP activity after exposure to the reparative cements NeoMTA-P, MTA-HP, PBS-HP, ProRoot, and Bio-C compared to the control group (CT). Only Biodentine was statistically similar to the CT. Data are presented as mean ± SD; one-way ANOVA, followed by multiple comparisons post-hoc test with Bonferroni’s test. Symbols on the chart indicate homogeneous groups by Bonferroni’s posttest. *p < 0.01, all groups versus CT; ^#^p < 0.001, NeoMTA-P versus all groups; **p < 0.02, MTA-HP versus all groups; ^##^p < 0.02, PBS-HP versus all groups; and ***p < 0.001, ZOE versus all groups.

Saos-2 cells exposed to the Bio-C (1078.6 thymolphthalein/min/L/OD ± 109.6), PBS-HP (1128.5 thymolphthalein/min/L/OD ± 69.1), NeoMTA-P (1301 thymolphthalein/min/L/OD ± 67), ProRoot (1159 thymolphthalein/min/L/OD ± 64.5) and MTA-HP (1205 thymolphthalein/min/L/OD ± 63.2) cements showed significant increases in ALP activity compared to the CT group. The ALP activity of Biodentine (1003 thymolphthalein/min/L/OD ± 57.8) was similar to that of the CT group (1000.7 thymolphthalein/min/L/OD ± 49.8) (Fig. 4).

### Mineralization assay

**Figures 5:**
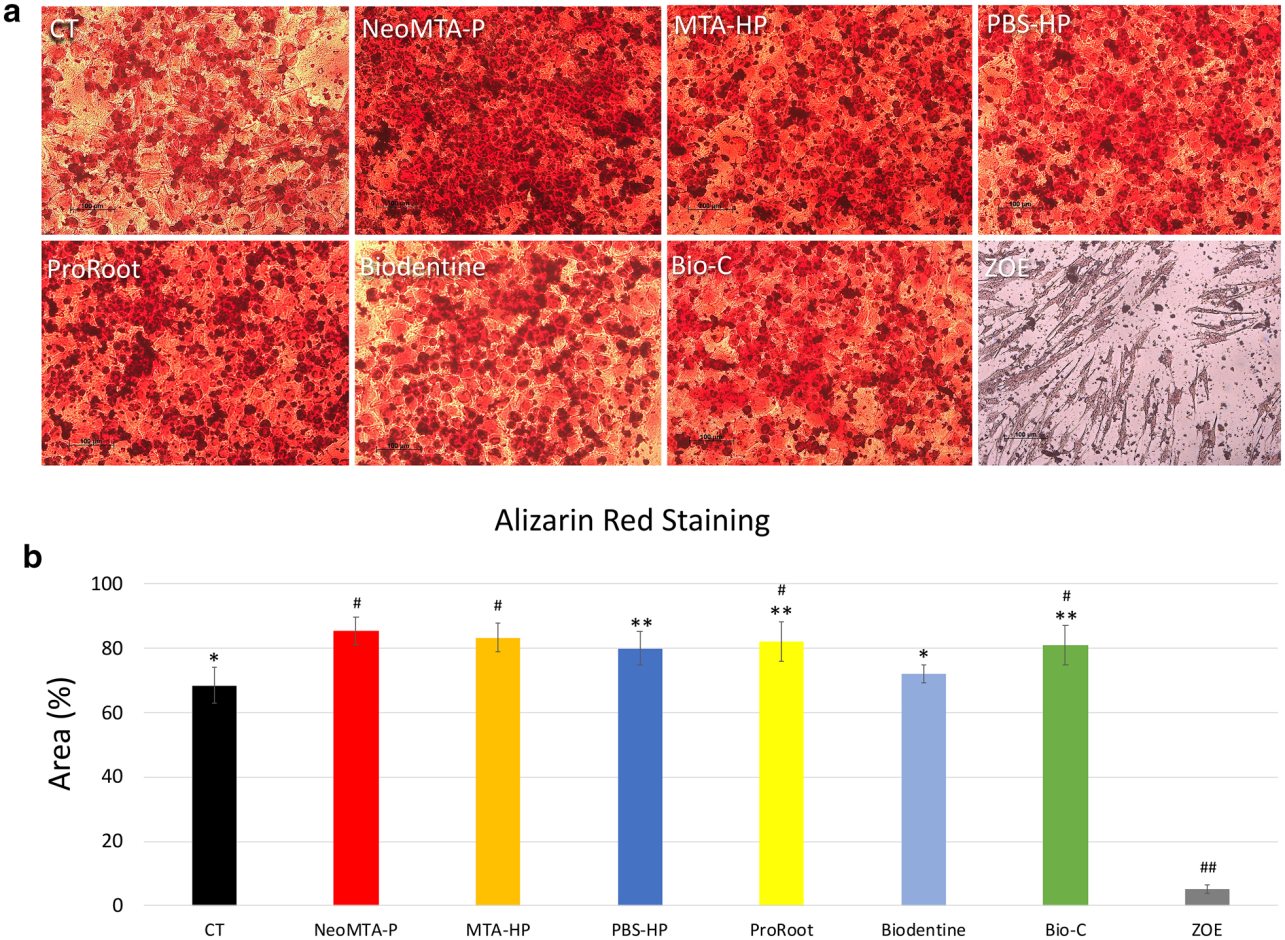
(**a**,**b**) Mineralization assay to detect calcium nodules and alizarin red staining (ARS). After 15 days of exposure, the representative images show the mineralized nodules in the NeoMTA-P, MTA-HP, PBS-HP, ProRoot, Biodentine, and Bio-C groups throughout the field (**a**). The ZOE group image shows almost no mineralization or morphological changes, and the cells appear shrunken (**a**). Statistical analysis showed significantly increased staining for all cement groups compared with the CT, except Biodentine and ZOE (**b**). Data are presented as mean ± SD; one-way ANOVA, followed by multiple comparisons post-hoc test with Bonferroni’s test. Symbols on the chart indicate homogeneous groups by Bonferroni’s posttest. *p < 0.001, all groups versus CT; ^#^p < 0.01, comparisons among cement groups; **p < 0.05, comparisons among cement groups; and ^##^p < 0.001, ZOE versus all groups.

The mineralization potential of the cells exposed to the six different reparative cements was verified using an alizarin red staining (ARS) assay and the results were compared to those of the control group (CT) and ZOE group (zinc-eugenol cement, a negative comparator). After 15 days in osteogenic media under cell culture conditions, the mineralization in all of the cement groups were significantly different from that in the CT (p < 0.05), except Biodentine (Fig. 5a,b). Mineralization staining (ARS) was impressive in the NeoMTA-P, MTA-HP, PBS-HP, ProRoot, and Bio-C groups (Fig. 5a). The NeoMTA-P images showed an upper layer rich in mineralization nodules (Fig. 5a). However, there was no significant difference in the percentage of mineralized areas between the NeoMTA-P, MTA-HP, PBS-HP, ProRoot, and Bio-C groups (Fig. 5b). The PBS-HP ARS percentage was significantly lower than that in the NeoMTA-P and MTA-HP groups (p < 0.05), but the cells continued to have high mineralization potential (ARS of approximately 80%) compared to the CT. The cells exposed to ZOE showed altered morphological characteristics and almost no staining (Fig. 5a). The ARS area in the ZOE group was significantly lower than that in the other groups (Fig. 5b).

## Discussion

The present study is the first to compare the biological properties of Bio-C, PBS-HP, MTA-HP, Biodentine, ProRoot, and NeoMTA-P in a culture of human osteoblastic cells. The calcium silicate cements showed different biological effects in Saos-2 cells, refuting our null hypothesis. In particular, Bio-C, PBS-HP, MTA-HP, and NeoMTA-P showed significantly low migration rates in the first 48 h of osteoblastic cell exposure. Bio-C treatment surpassed standard MTA-based cements with respect to osteocalcin gene expression. The cell migration and gene expression patterns indicated a shift toward osteogenesis after osteoblastic cell exposure to this endodontic repair material.

Biocompatibility and bioactivity are relevant properties that can lead the material to be the first choice for reparative and regenerative endodontic treatments. Saos-2 cells exposed to Bio-C and PBS-HP showed high viability rates, revealing the biocompatibility of these cements to human bone cells. Most likely, this biocompatibility is a consequence of the material's ability to maintain a basic pH in the surrounding environment and calcium ion (Ca^2+^) release^18^. The Ca^2+^ concentration in the cytoplasm is essential to mitochondrial activity, as evaluated by an MTT assay^19^. According to our results, ProRoot, MTA-HP, Neo MTA-P, and Biodentine can also be considered biocompatible with osteoblastic cells. However, a different study reported cytotoxicity to human apical papilla cells after exposure to ProRoot and Biodentine in a dose- and time-dependent manner^20^. ProRoot and Biodentine showed similar cytotoxicity in MG63 osteoblastic cells and human dental pulp cells^21,22^. The divergence of responses between these diverse cell lines highlights the relevance of cell culture studies. Studies in cell culture systems can predict the possibility of adverse effects, depending on the type of tissue exposed to the material. The setting time, direct tissue contacts, specificities of each cell line, optimal stiffness for cell migration, and elutes containing toxic elements from the cement composition can all influence the biocompatibility and bioactivity^23^. A study evaluating Biodentine, Bio-C Pulpo, TotalFill BC RRM (Root Repair Material), TheraCal LC, and ACTIVA BioACTIVE showed that the water-based cements had greater calcium ion release, which was associated with a more substantial antimicrobial effect but not enhanced biological activity^10^. The additives altered the hydration and leaching profile of the prototype cement. Microsilica inclusions resulted in decreased long-term calcium hydroxide formation associated with neutralized cytotoxicity and antibacterial activity^10^. For instance, ProRoot uses bismuth oxide as a radiopacifying agent, a compound that has been reported to present some level of cytotoxicity^24^. Biodentine contains brown iron oxide and CaCl_2_ to shorten the setting time^21^, which could also be responsible for some cytotoxic effects. The authors that observed a low viability rate for cells exposed to Biodentine posed two possible hypotheses: one was the effect of cell differentiation disallowing the cells to enter a proliferative phase, and the other hypothesis was due to cytotoxicity that impairs metabolism^20^. Their study used Alamar Blue to assess the viability of the cells^20^ and suggested that it would also be interesting to assess whether the bioactive compounds affected mitochondrial activity, which is precisely the principle of the MTT assay used in our study. Except for the cells in the NeoMTA-P, MTA-HP, and ZOE groups, the osteoblast-like cells showed mitochondrial dehydrogenase activity similar to that of the CT (by MTT assay). Together, the response of the osteoblastic cells to the material exposure by MTT assay, wound healing assay and the pattern of gene expression support the cell differentiation profile hypothesis instead of the cytotoxic effect. Cytotoxicity was observed only for ZOE as a comparator group, whose treatment led to substantial cell death and inhibition of osteogenic marker expression.

The wound healing experiment offered relevant information regarding the viability and migration of cells exposed to calcium silicate cements. The wound gap after 96 h of exposure was still open in all groups of cells exposed to these materials. However, no significant cellular morphological characteristic due to possible cytotoxic effects from NeoMTA-P, MTA-HP, PBS-HP, ProRoot, Biodentine, or Bio-C were observed. The cells seemed to proliferate on top of each other when exposed to the calcium silicate-based cements and formed cohesive clusters instead of migrating toward the wound, as observed in the control group. Curiously, the migration rates were very low during the first 24 and 48 h of exposure to the osteoblastic cells, except for in the Biodentine group at 48 h. Cell migration is recognized to be sensitive to mechanochemical environmental stiffness. Each cell type exhibits an optimum stiffness, at which migration reaches its maximum^25^. In the Bangasser et al*.* model, intracellular molecules such as myosin II transmit force to the external environment through actin filament bundles and compliant transmembrane molecular proteins^26^. This group studied the effects of substrate stress on cell spreading, collective cell durotaxis, and ex vivo cell migration as a function of adhesion molecule expression. It is possible that compounds released from calcium silicate cements into the environment affect F-actin flow, nonmuscle myosin II (NMII), and adhesion molecule expression in osteoblastic cells, contributing to or impairing their mechanical properties, a hypothesis to be further investigated^27^. NMII has been demonstrated to play an essential role in cell morphology, migration, and differentiation^27^. NMII generates forces that alter biochemical signaling by driving changes in interactions between actin-associated proteins that can ultimately regulate gene transcription and cytoskeletal organization and alter actomyosin contractility in stems^27^. Adhesion and migration rates are mechanical properties of osteoblasts that are important in the cell response to biomaterials and modulation of bone formation^26^. The mechanical properties, cytoskeletal structure, and motility of cells are known to reflect different cell differentiation stages^25,26^. Together, the wound healing results indicated that all of the tested calcium silicate-based cements affected the mechanical properties and motility of the osteoblastic cells to different extents.

Tissue stiffness should be considered when choosing a dental material for pulp or bone repair. For example, standard tissue stiffness varies from brain tissue to bone tissue^26^. In our study, the cell migration rates increased only after 72 h of exposure in both groups, which is indicative of the environmental stiffness of the osteoblastic cells exposed to Bio-C and NeoMTA-P. After 96 h of exposure to the Bio-C, PBS-HP, ProRoot, NeoMTA-P, and Biodentine cements, the cells presented a faster migration rate than the control group. A study with MTA-HP and NeoMTA-P on human pulp cells exposed to elutes showed similarly low migration rates and open wounds after 48 h without cytotoxic characteristics^28^. The activation of Ca^2+^ signaling pathways may explain osteoblastic cell behavior after exposure to calcium silicate cements. A high concentration of Ca^2+^ released from the cement possibly stimulated cell differentiation to the detriment of proliferation^10^. Evaluation of the water-based tricalcium silicate materials BCP, Biodentine, and TotalFill in 3T3 and hDPC cultures indicated that the inclusion of additives in hydraulic cements affected Ca^2+^ release to different extents, which in turn modified the biocompatibility and bioactivity of the leachates^10^. A study on an MTA-based cement showed dual modulation in human dental pulp cells through calcium-sensing receptors (CaSRs) triggered by Ca^2+^ and alkaline pH^29^. Cascade activation of the MTA-CaSR-phospholipase C pathway played an essential role in osteogenic differentiation by regulating the transcription of the osteocalcin (*BGLAP*), osteopontin (*SPP1*), and bone sialoprotein (*BNSP*) genes^29^.

The patterns of expression of *ALPL* (alkaline phosphatase), *BNSP*, and *BGLAP* divide osteoblastogenesis into 3 phases: the initial phase (*ALPL* overexpression), the intermediate phase (*BNSP* overexpression), and the advanced phase (*BGLAP* overexpression)^30^. In this study, osteoblastic cells exposed to Bio-C and MTA-HP showed the highest fold change in *BGLAP* expression (MTA-HP ~ BIO-C > ProRoot), which is indicative of the advanced phase of cell differentiation. MTA-based materials have been shown to induce osteoblastogenesis through the activation of *BGLAP* by transcription factor 6 (Atf6)^29^. Osteocalcin is the most abundant noncollagen protein in bone and an important mineralization marker. It is possible that osteocalcin regulates the mineralization rate^30^. *BNSP*, another marker of osteogenesis, was significantly overexpressed in the ProRoot, Bio-C, and NeoMTA-P groups (ProRoot > Bio-C ~ NeoMTA-P). In contrast, *ALPL* gene expression was inhibited in the Bio-C, MTA-HP, and ProRoot groups. Only the Biodentine group showed *ALPL* overexpression, a marker of initial differentiation^30^. A study with hDPSCs exposed to Biodentine and ProRoot indicated that the high pH and release of calcium ions could influence the differential expression of the osteogenesis markers *BGLAP* and *BMP-2*^9^. The alkaline phosphatase activity in osteoblastic cells exposed to the repair cement significantly increased after 5 days compared to the control group. Except for the Biodentine group, which showed no difference in ALP activity, the alkaline phosphatase results followed *ALPL* expression inhibition as negative feedback for Biodentine. Another study supported this hypothesis with Saos-2 cells exposed to MTA Plus^11^. PBS-HP was the cement that showed the highest migration rate in the first 24 h of cell exposure and the lowest fold change in gene expression. However, ALP activity was high in this cement group after 5 days of exposure. PBS-HP has a short setting time due to the pozzolan in its composition^15^, which probably leads to the bioavailability of Ca^2+^ in a time-dependent manner that interferes with the material’s potential to induce osteogenesis^9,10^. The results of our study, especially the osteogenic marker expression pattern and ALP activity, indicated an intermediate to advanced phase of osteoblastic differentiation in the NeoMTA-P, ProRoot, Bio-C, MTA-HP, and PBS-HP groups. These materials seemed to favor an immediate osteogenic effect on osteoblastic cells at the expense of cell migration, which corroborates the wound healing results. In contrast, the Biodentine group exhibited a pattern of initial osteoblast differentiation.

The mineralization potential of the osteoblastic cells after 15 days of exposure to NeoMTA-P, ProRoot, Bio-C, MTA-HP, and PBS-HP was consistent with the intermediate/late phases of differentiation. The presence of mineralization nodules stained with alizarin red in those groups was extensive. The ARS-stained areas were significantly higher in the NeoMTA-P, ProRoot, Bio-C, MTA-HP, and PBS-HP groups. The osteoblastic cells exposed to Biodentine had no differential pattern of mineralization when compared to the unexposed cells (CT). This result is consistent with the Biodentine early phase of differentiation, gene expression, and alkaline phosphatase activity. Saos-2 cells exposed to Biodentine and ProRoot in this study showed a different result than what was observed in human dental pulp cells in a different study^9^. Biodentine may be more suitable for pulp cells than for osteoblastic cells. After all, this material was developed to resemble dentine, the mineralized tissue surrounding the pulp cavity. It is important to remember that cells from diverse tissues present different characteristics, such as optimal stiffnesses, proliferation, calcium signaling responsiveness, and phases of differentiation. For example, a higher pH is the ideal environment for osteoblastic cells^11^. Tricalcium silicate-based cements are recognized for their high release of OH^-^, causing the environmental pH to increase^10^. The setting time can also explain the different results; we allowed the materials to set for 24 h before cell exposure, while in the dental pulp cell study, Biodentine and ProRoot were set for 3 h^9^. Complete setting and less toxic compound release could explain the best results from ProRoot in osteoblastic cells. A recent study evaluated ProRoot and Biodentine in the dental pulp of Wistar rats^31^. Their results showed that both materials presented a slight and reversible inflammatory reaction in the dental pulp. The authors also noticed an exacerbated induction of mineralization with Biodentine and argued that the use of this material could lead to the formation of pulp calcifications, which would be undesirable for regenerative endodontic treatment.

In conclusion, the potential among the calcium silicate materials in this study to induce osteogenesis was diverse. These materials seemed to induce different phases of cellular differentiation. The compounds released from the reparative cements probably created an environment rich in calcium ions, silica, and hydroxides that caused environmental stiffness around the osteoblastic cells, especially during the first 24 h of exposure. We believe that cement that immediately induces osteoblast differentiation is ideal for reparative and regenerative treatments because it would quickly stimulate a mineralized barrier. For instance, NeoMTA-P, Bio-C, PBS-HP, ProRoot, and MTA-HP would be more suitable for root canal perforations and apical surgeries. Cells exposed to Bio-C, PBS-HP, ProRoot, NeoMTA-P, and MTA-HP seemed to achieve advanced or intermediate phases of differentiation more quickly than those exposed to Biodentine. Biodentine may be more appropriate as a dentine substitute for indirect or direct pulp treatment. Particularly in pulp revascularization treatment, the new dentin bridge induced by the cement will favor pulp-like tissue regeneration underneath. Therefore, a cement with a higher potential to induce osteogenesis could be more attractive in regenerative endodontics; this hypothesis should inspire further evaluation in a clinical trial. Considering the putty formulation of Bio-C and the biological properties required for an endodontic reparative material, Bio-C and PBS-HP can be suitable options for ProRoot, MTA-HP, Biodentine, and NeoMTA-P. Several types of endodontic cements based on calcium silicate are available; each has a different composition and biological potential. The thought of adopting just one of them to use in all reparative or regenerative endodontics might be a mistake. After all, one cement has a better antibacterial effect while another can more effectively induce the differentiation of osteoblasts than the differentiation of pulp stem cells. This study can be a breakthrough in current clinical therapeutics for reparative endodontic treatments.

## Methods

### Culture of Saos-2 cells

Saos-2 human osteoblastic cells (ATCC HTB-85), a model of osteogenesis, were cultured as a monolayer in T-75 flasks (Corning, Union City, CA, USA) containing Dulbecco’s modified Eagle’s medium (DMEM), 10% fetal bovine serum (FBS), penicillin (100 IU/mL) and streptomycin (100 μg/mL) at 37 °C, 95% humidity, and 5% CO_2_ (all supplements, Gibco-BRL, Gaithersburg, MD, USA). After detaching with trypsin/EDTA (0.25%), Saos-2 cells were seeded at a density of 2 × 10^4^/well in 12-well plates (Corning, Union City, CA, USA) and cultured under the same conditions until adherence. The cells were then exposed to the cement samples in Transwells (0.4 µm permeable membranes, Corning Incorporated, Corning, NY, USA) for further experiments. Unexposed cells were the control group (CT). The experiments were repeated independently three times (n = 9/group). For the alizarin red staining (ARS) assay, the cells were exposed or not (CT) to the test materials in osteogenic media under the same conditions described above (osteogenic media: DMEM, 10% FBS, 100 IU/mL penicillin, 100 µg/mL streptomycin, 0.0023 g/mL β-glycerophosphate, 0.055 mg/mL l-ascorbate; Sigma Chemicals, St Louis, MO).

### Samples of endodontic cement

Bio-C, Biodentine, PBS-HP, MTA-HP, ProRoot, NeoMTA-P (Table 1), and zinc oxide-eugenol cement (ZOE, a negative control) were prepared according to the manufacturer’s recommendations under sterile conditions. The cement samples were placed in polypropylene molds (3 × 5 mm) and incubated for 24 h at 37 °C, 95% humidity, and 5% CO_2_ for complete setting before the experiment. They were then removed from the molds and placed in the Transwell inserts for exposure to Saos-2 cells in 12-well plates.

**Table 1:** Endodontic cement specifications.

Brand	Formulation	Initial setting time (min)	Composition	Manufacturer
ProRoot MTA	Powder-liquid	165	Powder: bismuth oxide, tricalcium silicate, dicalcium silicate, calcium dialuminate, and calcium sulfate. Liquid: sterile distilled water*	Dentsply Sirona Tulsa, OK, USA
Bio-C Repair	“Ready-to-use”	40	Calcium silicates, calcium aluminate, calcium oxide, zirconium oxide, iron oxide, silicon dioxide, and dispersing agent*	Angelus Londrina, PR, Brazil
PBS HP	Powder-liquid	5	Powder: calcium oxide, calcium carbonate, magnesium oxide, dicalcium silicate, aluminum oxide, sodium oxide, potassium oxide, and pozzolan (high level of silica oxide and aluminum highly reactive with calcium hydroxide). Liquid: distilled water*	CIMMO MJS Ltda Pouso, Alegre-MG Brazil
Biodentine	Powder-liquid	12	Powder: tricalcium silicate, zirconia oxide, calcium oxide, calcium carbonate, yellow and red pigment, brown iron oxide. Liquid: calcium chloride dihydrate, purified water*	Septodont Saint-Maur-des-Fossés, France
NeoMTA Plus	Powder-gel	< 60	Powder: tricalcium silicate, dicalcium silicate, tantalum oxide, tricalcium aluminate, calcium sulfate, and plaster. Liquid: water-based gel and thickening agents and water-soluble polymers*	Avalon Biomed Houston, TX, USA
MTA Repair HP	Powder-liquid	15	Powder: tricalcium silicate, dicalcium silicate, tricalcium aluminate, calcium oxide, calcium tungstate. Liquid: water and plasticizer*	Angelus Londrina, PR, Brazil

*Manufacturer information.

### MTT assay

After 24 h of exposure to the cement pellets, the medium was replaced by DMEM without FBS containing 0.55 mg/mL MTT (Sigma Chemicals, St Louis, MO, USA). The plates were incubated for an additional 4 h under the same conditions already described to allow the mitochondrial dehydrogenases of viable cells to transform the yellow MTT salt into purple formazan crystals. After that, each well was washed with 1 mL of phosphate buffer (PBS 1×), and the formazan was solubilized with 500 μL of acidic isopropyl alcohol (isopropyl alcohol with 0.04 N HCl). A total of 100 μL from each sample was transferred to 96-well plates, and the optical density at 590 nm (OD 590 nm) of each well was measured with a microplate reader (SpectraMax M3, Molecular Devices, San Jose, California, USA). The viability rates of the cells exposed to the cement samples and the control group were calculated according to the following formula: viability rate = (OD of the sample × 100)/OD of CT.

### Wound healing

When the Saos-2 cells were 95% confluent, we made a 500 μm wide wound at the bottom of each well in 12-well plates, and the medium was renewed. The experiment was performed in triplicate for cell exposure to each cement sample in Transwell inserts and was repeated three times independently. A total of 30 fields were photographed per group using an Axio Observer Z1 microscope (Carl Zeiss, Jena, Germany). Digital images were taken at different exposure times: 0 h, 24 h, 48 h, 72 h, and 96 h. The microscopic images (n = 30/group) were processed using ImageJ 1.52K Software (National Institutes of Health, NIH, Bethesda, Maryland, USA). The cell covered area (%) was calculated with ImageJ following the equation: $\mathrm{A}\left(\mathrm{\%}\right)=\frac{\mathrm{Ac}\times 100}{\mathrm{Ag}},$ where Ac is the cell covered area of the gap and Ag is the total area of the gap. The migration rate (µm/h) was calculated as described by Jonkman et al*.*^32^. According to the authors, we first measured the gap area for each frame in the wound healing experiment and then plotted the gap area as a function of time, deriving the cell migration rate (Vm) and the time in hours (h) to close half of the gap (t1/2 gap). We obtained a general equation for a line, in which the intercept is the gap area at the start of the experiment. The cell sheet migration rate (Vm) is the average velocity at which the cells collectively move into the gap created in the *wound healing* assay. The slope is equal to dA/dt, where the area A (µm^2^) is the width of the gap (*w*) times the length of the gap (*l*). Assuming that the gap is much longer than the field of view so that the cells do not migrate from the edges, the length is constant^32^. Considering that the width closes at twice the rate of cell migration, this gives a cell migration rate of $\mathrm{Vm}=\frac{|\mathrm{Slope}|}{2\mathrm{l}}=\frac{|\mathrm{dA}/\mathrm{dt}|}{2\mathrm{l}}$.

### Real-time PCR (qPCR)

The same cells observed in the wound healing assay were collected in TRIzol (Thermo Fisher Scientific, Waltham, Massachusetts, USA) after 96 h of exposure. Total RNA was extracted according to the TRIzol manufacturer's instructions. From each sample, 2 μg of RNA was reverse transcribed in cDNA (QuantiTect Kit, Qiagen, Hilden, Germany) and used as a template for real-time PCR (qPCR) to evaluate the osteogenesis markers bone sialoprotein or secreted phosphoprotein 1 (*BNSP*), alkaline phosphatase (*ALPL*) and osteocalcin or bone gamma-carboxyglutamate protein (*BGLAP*). Glyceraldehyde-3-phosphate dehydrogenase (*GAPDH*) was the reference gene. The reactions were carried out in a Rotor-Gene Q system (Qiagen, Hilden, Germany) and included SYBR-Green PCR Master Mix 1X (Bio-Rad, Hercules, California, USA), 100 ng of cDNA, and 0.25 µM of each forward and reverse primer (Exxtend, Paulínia, SP, Brazil) in a final volume of 20 µL. Thermal cycle conditions: 95 °C for 5 min, 40 cycles of denaturation at 95 °C for 5 s and priming, synthesis plus fluorescence reading at 60 °C for 15 s. The fold change in expression followed the ∆∆Ct method (fold = 2^−(∆∆Ct ± stdv)^). The forward primer (FP) and reverse primer (RP) were *BNSP* (L24757.1), FP = 5′TGCATCGAAGAGTCAAAATAGAGG3′ and RP = 5′GAGGATAAAAGTAGGCATGCTTG3′; *BGLAP* (NM_199173), FP = 5′CAGCGAGGTAGTGAAGAGAC3′ and RP = 5′TGCTTGGACACAAAGGCTG3′;

*ALPL* (NM_000478.4), FP = 5′AAGCACTCCCACTTCATCTG3′ and RP = 5′TTGTTCCTGTTCAGCTCGTAC3′; and *GAPDH* (NG_007073), FP = 5′TGAAGGTCGGAGTCAACG3′ and RP = 5′TGGGTGGAATCATATTGGAAC3′.

### Alkaline phosphatase activity

The enzymatic activity of alkaline phosphatase (ALP) was evaluated using a colorimetric kit (Labtest, Lagoa Santa, MG, Brazil) as described in a different study^33^. Briefly, after 5 days of exposure, Saos-2 cells were immersed in 500 µL of sodium lauryl sulfate (1 mg/mL, Sigma Chemicals, St. Louis, MO, USA) for 30 min at room temperature, and 50 μL of sample from each group was added to the kit components according to the manufacturer's instructions. The absorbance (OD = 570 nm) was measured with a microplate reader (SpectraMax M3, Molecular Devices, San Jose, California, USA). The ALP activity was expressed as μmol of thymolphthalein/min/L/OD of viable cells for normalization^33^.

### Alizarin red staining (ARS)

After 15 days of cell exposure to the cements in osteogenic media (n = 6/group), the culture wells were washed three times for 5 min each with 1× PBS and fixed in 10% (v/v) formaldehyde (Sigma) at room temperature for 15 min. The monolayers were then washed twice with sterile distilled water (dH_2_O) for 5 min each time. After that, 1 mL of 2% ARS, pH 4.1 (Sigma) per well was added to the plate wells, and the samples were incubated again at room temperature for 20 min to expose the mineralized nodules to the dye. The wells were finally washed three times with 2 mL of dH_2_O. The stained fields were observed using an inverted microscope (Axiovert 100, Carl Zeiss, Jena, Germany) at 40× magnification and photographed. The digital images were processed using ImageJ 1.45 software (National Institutes of Health, NIH, USA), and the percentage of stained area was calculated according to ImageJ guidelines. Briefly, all images were converted to RGB (red, green, blue), the threshold of the red channel was adjusted in ImageJ taking the images of the CT (control) as a parameter, and the percentage of stained area in all groups were measured under the previously defined threshold (n = 12 images/group).

### Statistical analysis

The MTT, ALP, qPCR, and wound healing assays and ARS data were tested for normal distribution using the Shapiro–Wilk W test (sample sizes between 3 and 2000). All data presented a normal distribution. MTT, ALP, qPCR, and ARS data were then subjected to one-way ANOVA, and the wound healing data to two-way ANOVA, where the response variable was migration rate, and the two factors were time and the material group. Sample size calculations were established using the means and within-group variance based on results from other studies^11,17^, with an alpha of 5% and 80% power (“F test for group effect” and “F test for row effect”, considering one-way ANOVA and two-way ANOVA, respectively). Differences between groups were established with Bonferroni’s posttest for all variance analysis, with p < 0.05 (all in Stata/IC 15.1, StataCorp, College Station, TX, USA).

### Ethical approval

This article does not contain any studies with human participants or animals performed by any of the authors.

### Informed consent

For this type of study, formal consent was not required.
